# Development and epithelial organisation of muscle cells in the sea anemone *Nematostella vectensis*

**DOI:** 10.1186/1742-9994-11-44

**Published:** 2014-06-18

**Authors:** Stefan M Jahnel, Manfred Walzl, Ulrich Technau

**Affiliations:** 1 Department of Molecular Evolution and Development, Centre for Organismal Biology, Faculty of Life Sciences, University of Vienna, Althanstrasse 14, 1090 Wien, Austria; 2 Department of Integrative Zoology, Centre for Organismal Biology, Faculty of Life Sciences, University of Vienna, Althanstrasse 14, 1090 Wien, Austria

## Abstract

**Introduction:**

*Nematostella vectensis*, a member of the cnidarian class Anthozoa, has been established as a promising model system in developmental biology, but while information about the genetic regulation of embryonic development is rapidly increasing, little is known about the cellular organization of the various cell types in the adult. Here, we studied the anatomy and development of the muscular system of *N. vectensis* to obtain further insights into the evolution of muscle cells.

**Results:**

The muscular system of *N. vectensis* is comprised of five distinct muscle groups, which are differentiated into a tentacle and a body column system. Both systems house longitudinal as well as circular portions. With the exception of the ectodermal tentacle longitudinal muscle, all muscle groups are of endodermal origin. The shape and epithelial organization of muscle cells vary considerably between different muscle groups. Ring muscle cells are formed as epitheliomuscular cells in which the myofilaments are housed in the basal part of the cell, while the apical part is connected to neighboring cells by apical cell-cell junctions. In the longitudinal muscles of the column, the muscular part at the basal side is connected to the apical part by a long and narrow cytoplasmic bridge. The organization of these cells, however, remains epitheliomuscular. A third type of muscle cell is represented in the longitudinal muscle of the tentacle. Using transgenic animals we show that the apical cell-cell junctions are lost during differentiation, resulting in a detachment of the muscle cells to a basiepithelial position. These muscle cells are still located within the epithelium and outside of the basal matrix, therefore constituting basiepithelial myocytes. We demonstrate that all muscle cells, including the longitudinal basiepithelial muscle cells of the tentacle, initially differentiate from regular epithelial cells before they alter their epithelial organisation.

**Conclusions:**

A wide range of different muscle cell morphologies can already be found in a single animal. This suggests how a transition from an epithelially organized muscle system to a mesenchymal could have occurred. Our study on *N. vectensis* provides new insights into the organisation of a muscle system in a non-bilaterian organism.

## Introduction

Muscles are present in all metazoans except sponges and placozoans. Their emergence marks an important step in evolution because it allows organisms to disperse, escape, hunt and explore new habitats. Muscle cells are a major derivative of the mesoderm in Bilateria, but can also be found in two non-bilaterian phyla, the Ctenophora and the Cnidaria. The diploblastic Cnidaria are of particular interest for understanding the evolution of key bilaterian traits because, they are considered to be the sister group of the Bilateria [1,2] and therefore occupy a crucial phylogenetic position. Cnidarian polyps generally have smooth muscles, yet medusae also have striated muscles [3]. The striking structural similarity of striated muscles in Cnidaria and Bilateria has led to the suggestion that striated muscles of Cnidaria and Bilateria are homologous [4]. These authors extended their arguments by proposing that cnidarians are reduced Mesodermata [4]. However, a recent phylogenomic study tracing the evolutionary origin of all muscle components known from model bilaterians revealed the absence of several crucial muscle proteins from the genome of non-bilaterian organisms as well as the bilaterian lineage-specific innovations of other crucial muscle proteins [5]. These phylogenetic and expression analyses led to the conclusion that striated muscles evolved convergently in cnidarians and bilaterians, on the basis of ancestral proteins, which predate the divergence of animals [5]. Furthermore, several key myogenic transcription factors such as MyoD and MRFs (myogenic regulatory factors) have not been identified in cnidarians. This raises questions of how muscles in cnidarians develop and how they are structured.

In recent years, *N. vectensis*, a representative of the Anthozoa, has been established as an important model for studying embryology, phylogenetic relationships, comparative genomics and the origin of triploblasty [6-8]. This makes it a promising addition to the existing group of cnidarian model systems. Gene expression and functional studies have led to a much better understanding of the molecular regulation of embryogenesis and larval development during the last decade. Nonetheless, our knowledge about the anatomy and cellular composition of this new model organism remains poor. Often, not even the cell types that account for a highly specific gene expression pattern are known. In particular, the adult stage is poorly understood. Hence, as *N. vectensis* continues to develop into a major cnidarian model organism, we need to reach a deeper understanding of the composition, connections and differentiation kinetics of the different cell types at various developmental stages.

Frank and Bleakney [9] investigated the general anatomy of *N. vectensis* at a histological level, yet the level of resolution and the detail of analysis did not enable conclusions to be drawn about the development and precise cellular composition of the various cell types.

Here, we present a detailed anatomical description of the muscular system of *N. vectensis* using histology, electron microscopy, confocal microscopy and transgenic lines, specifically expressing reporter genes in retractor muscles of the column and tentacles. We show that muscle cells display different levels of epithelial organization, dependent on their position in the organism. They vary from an epitheliomuscular organisation to a basiepithelial muscle cell, which has lost all apical cell-cell junctions and subsequently is positioned at the base of the epithelium. Our data suggest that epitheliomuscular cells can be highly modified to comply with spatial constraints. Based on these results we interpret the different modes of epithelialization to represent intermediate steps of detachment from the epithelium, which finally would enable muscle cells to become located between the ectoderm and endoderm.

## Methods

### Animal culture

Animals were cultured and spawning was induced as described elsewhere [10,11]. In brief, animals were kept at 18°C, predominantly in the dark, and spawning was induced by raising the temperature to 24°C in the presence of light for 10 h.

### Histology

Adult animals were anesthetized with a few drops of 7% MgCl_2_ for 30 min and fixed in 4% PFA (paraformaldehyde) in PBT (phosphate buffered saline, 0.2% Triton) overnight, washed in PBT, dehydrated in ethanol and methyl benzoate and embedded in paraffin. Sections (7 μm) were stained with Azan.

### Transmission electron microscopy

Samples were put on ice and anesthetized as described above with MgCl_2_ (primary polyps, adults) and subsequently fixed with 2.5% glutaraldehyde in 0.1 M cacodylate buffer (pH 7.2) for 1 h (planulae, primary polyps) or overnight (adults) at 4°C. After fixation, samples were either stored in 1.25% glutaraldehyde in 0.1 M cacodylate buffer (pH 7.2) at 4°C or processed immediately. Then they were washed in the same buffer used for fixation. Samples were postfixed in 1% OsO_4_ in 0.1 M cacodylate buffer (pH 7.2) for 30 min and washed with 0.1 M cacodylate buffer (pH 7.2). Thereafter they were dehydrated through a graded series of ethanol and acetone and embedded into the Low Viscosity Resin (Agar) following sectioning using routine techniques. After staining with watery solutions of uranylacetate (20 min) and lead citrate (10 min), sections were examined with a Zeiss Libra 120 transmission electron microscope.

### Phalloidin stainings

Animals were anesthetized as described above and fixed in 4% PFA in PBT overnight at 4°C and subsequently washed in the same buffer. Permeabilization was enhanced by putting embryos into ice-cold (−20°C) acetone for 7 min followed by thoroughly washing in PBT. Subsequently samples were incubated in Phalloidin-Alexa 488 (Invitrogen) (3 μl/100 μl PBT) for at least overnight at 4°C in the dark, washed in PBT and mounted on glass slides in Vectashield.

### Cryosectioning

Stained animals were cut into smaller pieces and incubated in OCT infiltration solution (20% OCT compound (Sakura), 25% sucrose in PBS) overnight, followed by putting them in a drop of 80% OCT (80% OCT, 25% sucrose in PBS) on a glass slide. A plastic mold was placed over them and filled with two additional drops of 80% OCT. After orientation the slide was placed on a metal block (precooled in liquid nitrogen) and immediately filled with 100% OCT. The solid frozen samples were stored at −20°C or processed immediately. Sections (14 μm) were cut in a Leica CM3050S cryostat at −24°C and collected on warm (RT) Superfrost Ultra Plus slides (Thermo Scientific). After a drying step (RT, overnight in the dark) the slides were placed in PBS for 5 min to wash away the embedding medium and mounted immediately in Vectashield.

### Antibody staining

Samples were anesthetized (primary polyps) and fixed in 4% PFA in PBT for 6 h, washed in PBT and blocked in blocking buffer (80% PBT, 20% sheep serum, 1% bovine serum albuminum) for 2 h at room temperature. They were then incubated in primary antibody (rat, anti-RFP; Chromotek) 1:400 in blocking buffer overnight at 4°C, subsequently washed in PBT and incubated in secondary antibody (goat, anti-rat, DyLight 549; Jackson) 1:500 in blocking buffer containing phalloidin (3 μl/100 μl) for 3 h at room temperature. After an additional washing step, samples were mounted on glass slides in Vectashield.

## Results

**Figure 1:**
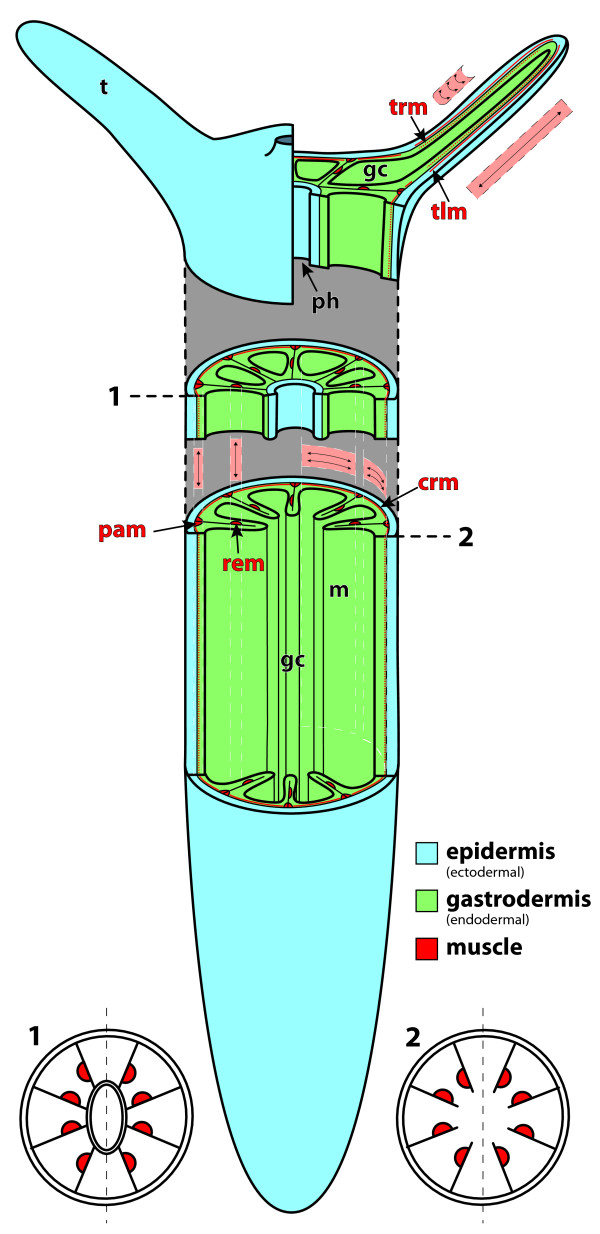
**The muscle system of *N. vectensis*.** Schematic overview of the muscle system in the adult polyp. The columnar musculature consists of the longitudinally orientated parietal and retractor muscle located in each of the eight mesenteries and a columnar ring muscle: All are of endodermal origin, the latter spanning the whole column between parietal muscles. In the tentacle an epidermal longitudinal muscle antagonises a gastrodermal ring muscle. Corresponding cross-sections of the polyp through the pharynx **(1)** and the subpharyngeal **(2)** region are shown to demonstrate the internal bilateral symmetry based on the arrangement of retractor muscles. crm, column ring muscle; gc, gastric cavity; m, mesentery; pam, parietal muscle; ph, pharynx; rem, retractor muscle; t, tentacle; tlm, tentacle longitudinal muscle; trm, tentacle ring muscle.

**Figure 2:**
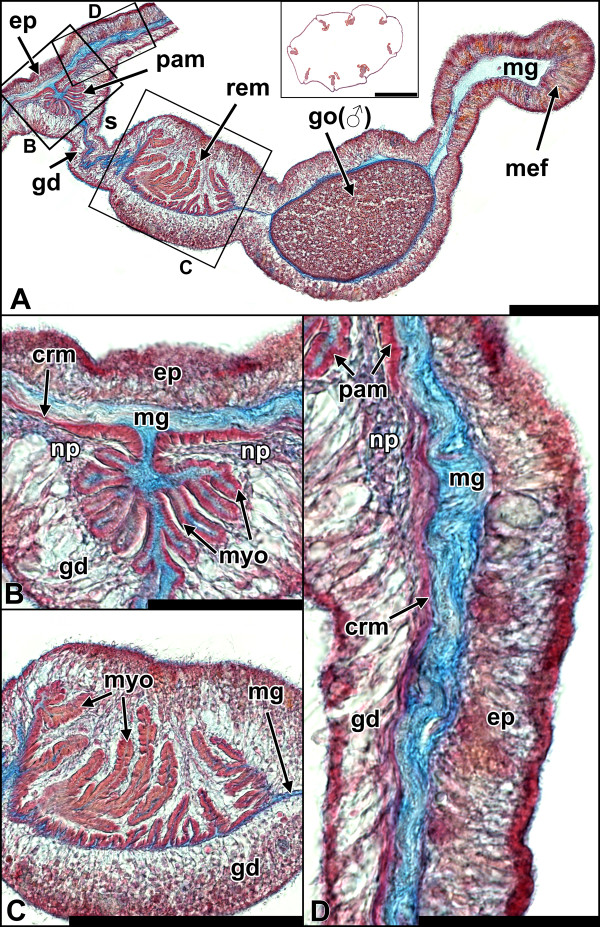
**Histology of the muscle system of *N. vectensis*.** All sections are cross-sections. **A** Architecture of a single mesentery. The parietal muscle is located at the base of the mesentery, followed by a more distal retractor. Between the retractor muscle and the mesenterial filament at the tip of the mesentery, a gonad, embedded into the mesoglea, is formed. Inlet: Cross-section through the subpharyngeal region showing all eight mesenteries. **B** Detail of **A**. The parietal muscle shows a typical bilateral folding of the mesoglea, on which the myonemes of the muscle cells attach. The muscle consists of two contiguous sheets of myonemes, which merge into the ring muscle laterally. Two distinct neural plexi are visible on either side next to the parietal muscle. **C** Detail of **A**. In the retractor muscle the mesoglea folds and branches only to one side. This folding is generally more pronounced than in the parietal muscle. **D** Detail of **A**. The ring muscle lies between the parietal muscle of each mesentery. Similarly, the myonemes are located at the base of the cells. ep, epidermis; gd, gastrodermis; go, gonad; mef, mesenterial filament; mg, mesoglea; myo, myoneme; np, neural plexus; pam, parietal muscle; rem, retractor muscle; crm, column ring muscle; s, stalk. Scale: **A, C**: 100 μm; **B, D**: 50 μm; inlet: 1 mm. All sections are stained with Azan.

**Figure 3:**
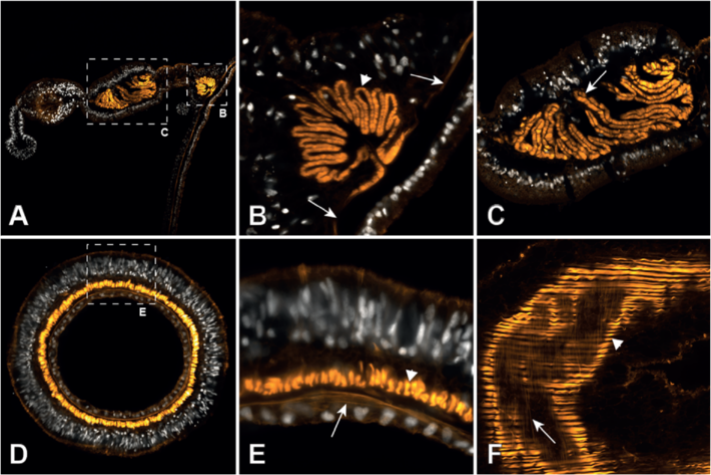
**Muscles of the adult polyp revealed by F-actin staining with phalloidin. A** Cross-section of a whole mesentery with proximal parietal muscle and distal retractor muscle. **B** Detail of **A**. Parietal muscle. Note the transition of the columnar circular muscle (arrows) into the longitudinally orientated parietal muscle (arrowhead). **C** Detail of **A**. Retractor muscle (arrow). **D** Cross-section of a tentacle. **E** Detail of **D**. The well-formed longitudinal muscle is located basiepithelially in the epidermis (arrowhead), whereas the circular muscle is weakly developed and located in the gastrodermis (arrow). **F** Single longitudinal optical section of a tentacle showing the strong longitudinal muscle filaments (arrowhead) overlying those of the circular muscle (arrow). white: DAPI, orange: phalloidin.

**Figure 4:**
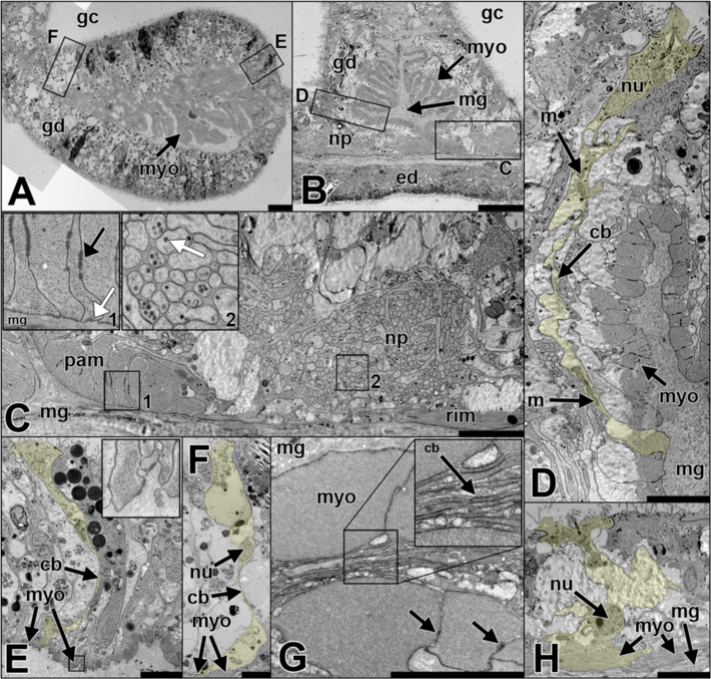
**Epithelial organization of the columnar muscles.** All sections are cross-sections **A** Overview of the retractor muscle region. **B** Overview of the parietal muscle region. **C** Detail of **B**. A distinct neural plexus consisting of numerous neurites is located next to the parietal muscle and close to the transition zone to the ring muscle. Inlet 1: spot-like adherens junctions between the basal part (black arrow) connect muscle cells to each other. Focal adhesions (white arrow) connect the cells to the mesoglea. Inlet 2: Detail image of neurites containing neural vesicles (white arrow). **D** Detail of **B**. Parietal muscle cells consist of a cell body involved in building up the epithelium. Via cytoplasmic bridges they are connected to the myofilament-containing basal part of the cell, adjacent to the mesoglea (cell highlighted in yellow); this yields a consistent sheet of myonemes. **E** and **F** Details of **A**. Muscle cells (highlighted in yellow) remain epithelial at least in the proximal and distal boundaries of the retractor muscle. **E** (inlet): Focal adhesion between muscle cell and mesoglea. **G** Detailed image of well-formed myonemes in the central region of the retractor muscle. Thin cytoplasmic bridges (inlet, black arrow) are projected by every cell. Basal parts of the cells are connected by spot-like adherens junctions (black arrows). **H** Detailed image of a ring muscle cell showing its participation in building up the epithelium. The myofilaments are located at the basal part of the cell. cb, cytoplasmic bridges; gc, gastric cavity; gd, gastrodermis; m, mitochondrium; mg, mesoglea; myo, myoneme; np, neural plexus; nu, nucleus; pam, parietal muscle; rim, ring muscle. Scale: **A, B**: 20 μm, **C-F, H**: 5 μm, **G**: 2 μm.

**Figure 5:**
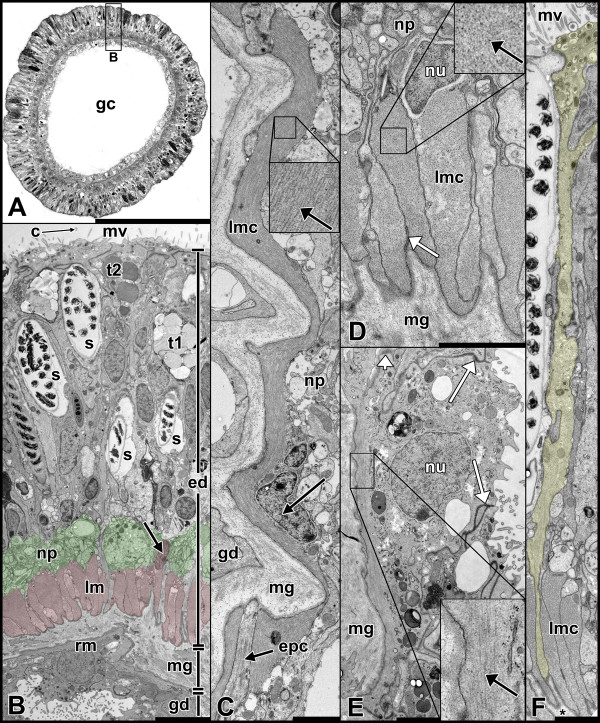
**Organization of the tentacle muscle system in an adult polyp. A** Tentacle cross-section. **B** Detail of **A**. Typical appearance of a tentacle epithelium. Longitudinally orientated muscle cells are situated side by side at the base of the epidermis and are connected to the mesoglea (highlighted in red). A neural plexus (highlighted in green) is located on top of the muscle layer. Epithelial cells are still connected to the mesoglea by thin processes, which find their way through the discontinuous sheet of muscle cells. In the gastrodermis the epithelial muscle cells are oriented circularly. **C** Longitudinal section of a longitudinal muscle cell pointing out the basiepithelial organization. Note the close location of the nucleus to the myofilaments. Inlet: parallel arrangement of thick myofilaments (arrow). **D** Detail of a cross-sectioned longitudinal muscle cell with adjacent nucleus. White arrow: spot-like adherens junction connecting muscle cells to each other. Inlet: Thick myofilaments (black arrow) are distributed irregularly between thin ones. **E** Ring muscle cell of the gastrodermis (cross-section) with myofilaments located at the base of the cell adjacent to the mesoglea. White arrow: Belt-like apical junctional complex. Inlet: In comparison to the longitudinal muscle the myofilaments of the ring muscles are weakly developed. Black arrow: Thick myofilament. **F** Epithelial cell (highlighted in yellow) spanning from the external surface to the mesoglea through the sheet of longitudinal muscle cells. Asterisk denotes mesoglea. c, cilium; ed, epidermis; epc, epithelial cell; gc gastric cavity; gd; gastrodermis; lm, longitudinal muscle layer, lmc, longitudinal muscle cell; mv, microvilli; np, neural plexus; nu, nucleus; muscle; rm, ring muscle layer; s, spirocyst; t1, type 1 gland cell with electron-light vesicles; t2, type 2 gland cell with electron-dense vesicles. Scale: **A**: 50 μm, **B**: 5 μm, **C-F**: 2 μm.

**Figure 6:**
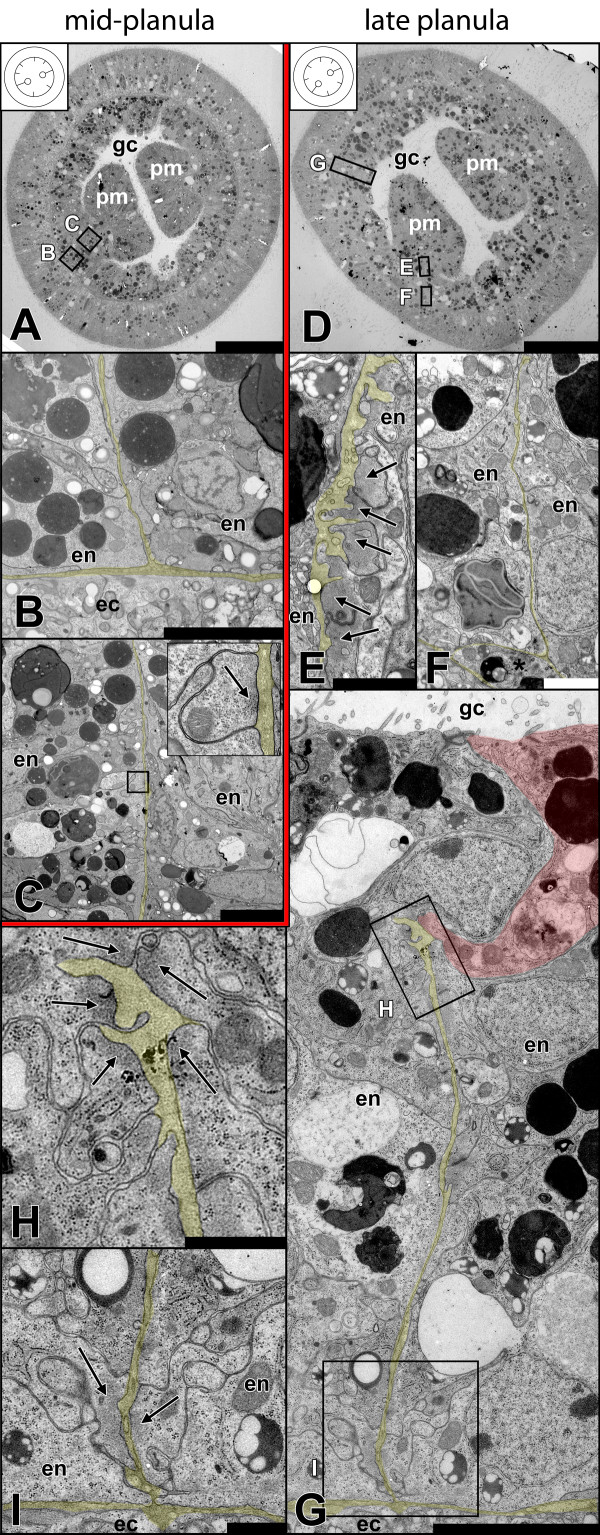
**Development of columnar longitudinal muscles in mid and late planula. A** Mid-planula (~4 d), overview. The first two emerging mesenteries (primary mesenteries) are formed opposite each other. Note that the location of the secondary mesenteries is already set at this stage (inlet). **B** Detail of **A**. The base of the mesentery (corresponding to the future site of parietal muscle formation) shows no distinct accumulation of myofilaments. **C** Detail of **A**. Single cell (inlet, black arrow: myofilaments) showing first sign of muscle formation. **D** Late planula (~5 d), overview. Primary mesenteries gradually shift to one side. **E** Detail of **D**. The developing retractor muscle in the primary mesentery can already be identified. Myonemes are formed exclusively on one side of the mesoglea. Black arrows: accumulating myofilaments. **F** Detail of **D**. Basal part of the primary mesentery. No myofilaments have accumulated yet. Note an amoeboid cell at the branching of the mesoglea, which could be detected occasionally (asterisk). **G** Detail of **D**. Secondary mesentery. Future retractor muscle cell highlighted in red. **H** Detail of **G**. Retractor muscle cells forming at the tip of the mesentery (in contrast to the primary mesentery), having no bias to one side at this stage. Black arrows: accumulating myofilaments. **I** Detail of **G**. Cells at both sides of the base of the mesentery start to accumulate myofilaments (black arrows) adjacent to the mesoglea. All sections are cross-sections of the subpharyngeal region. For easier understanding the mesoglea of all close-ups is highlighted in yellow. Inlets in A and D indicate the location of primary (lines with circles) and secondary mesenteries (lines without circles). ec, ectoderm; en, endoderm; gc, gastric cavity; pm, primary mesentery. Scale: **A, D**: 50 μm; **B, C, G**: 5 μm; **E**, **F**: 2 μm; **H, I**: 1 μm.

**Figure 7:**
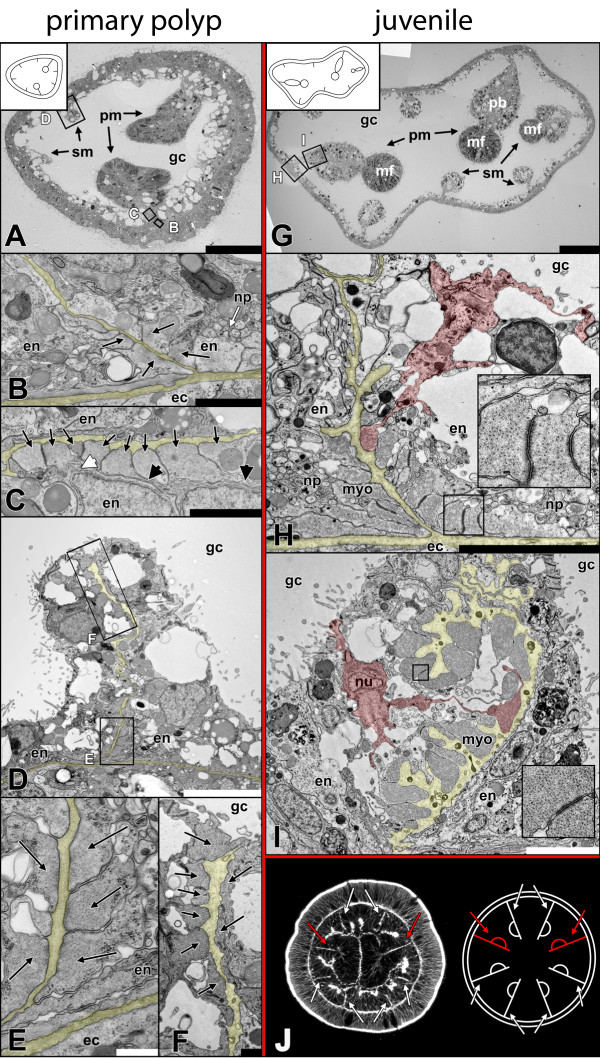
**Development of columnar longitudinal muscles in primary polyps and juveniles. A** Early primary polyp (~5 d), overview. **B** Detail of **A**. Cells located next to each other at the base of the mesentery increasingly accumulate myofilaments (black arrows) in their basal part. **C** Detail of **A**. Some retractor muscle cells (long black arrows) start to constrict the basal from the apical part of the cell, leaving behind thin cytoplasmic bridges (short black arrows), while in some the nucleus still lies near the myoneme (short white arrow). **D** Detail of **A**. Secondary mesentery. **E** Detail of **D**. As in primary mesenteries more cells accumulate myofilaments (black arrows) at their bases. **F** Detail of **D**. In the secondary mesenteries, myonemes (black arrows) are still located at the tip of the mesentery, but are more pronounced on one side. **G** Juvenile (~3 months) polyp, overview. **H** Detail of **G**. Despite the constriction of the myoneme, parietal muscle cells retain an epithelial organization (single cell highlighted in red). Inlet: Arrangement of myofilaments and adherens junctions between myonemes. **I** Detail of **G**. In the retractor muscle the sheet of myonemes starts to fold. Muscle cells are still epithelial (single cell highlighted in red). Inlet: Arrangement of myofilaments and adherens junctions between myonemes. **J** Optical cross-section of a mid-planula. The orientation of the retractor muscle is species-specific and predetermined for every mesentery. This allows predicting the future side of the retractor muscle in all mesenteries, as soon as all mesenteries have emerged. Red arrows: primary mesenteries, white arrows: secondary mesenteries. ec, ectoderm; en, endoderm; gc, gastric cavity; pb, proximal bulge; pm, primary mesentery; sm, secondary mesentery; mf, mesenterial filament, myo, myoneme; np, neural plexus; nu, nucleus. Scale: **A, G**: 50 μm; **B, C**: 2 μm; **D, H,** I: 5 μm, **E**: 500 nm; **F**: 1 μm.

**Figure 8:**
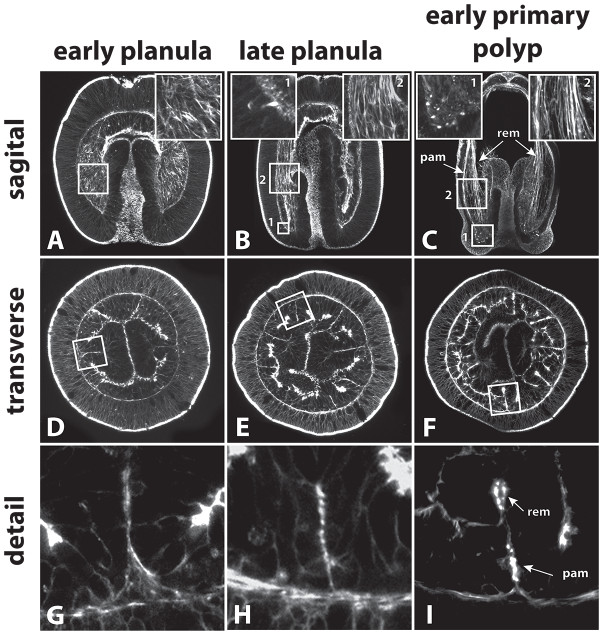
**Condensation of actin filaments during the differentiation of muscle cells. A, D, G**: Early planula. F-actin is weakly pronounced in the mesenteries but not orientated uniformly. **B, E, H**: In the late planula, F-actin becomes increasingly accumulated and oriented along the oral-aboral axis adjacent to the mesoglea. Moreover, future tentacle cells become enriched in F-actin (**B,** inlet 1). **C, F, I**: Early primary polyps show distinct longitudinal muscle strands and increased F-actin accumulation in tentacle muscle cells (**C,** inlet 1). The parietal muscle and retractor muscle can clearly be identified. Images in **A** (6 sections), **B** (8 sections) and **C** (20 sections) constitute maximum projections. pam, parietal muscle; rem, retractor muscle.

**Figure 9:**
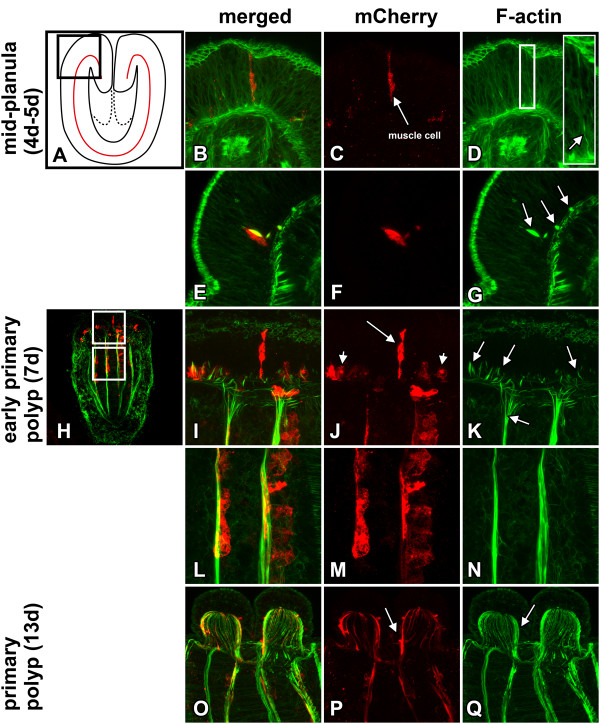
**Differentiation of tentacle longitudinal muscle cells in a muscle specific transgenic (MHC::mCherry) reporter line**. F-actin was stained with phalloidin (green), mCherry with an α-RFP antibody (red). **A-G** Optical longitudinal sections of mid-planula stages (4–5 d) in the future tentacle bulb region. A Overview. **B-D** Maximum projection of six layers. An early undifferentiated tentacle muscle cell (labelled by the α-RFP antibody) spans throughout the ectodermal epithelium and already shows a weak accumulation of actin filaments (**D,** inlet). E-G Maximum projection of seven layers. Tentacle muscle cells lose connection to the apex of the epithelium. Actin filament accumulation at the base of the epithelium increases in several cells (**G,** arrows). **H-N** Optical longitudinal sections of early primary polyp stages (7 d). Maximum projection of 21 layers. Different stages of detachment of tentacle longitudinal muscles from the epithelium co-occur at the same stage (**J,** long arrow: epithelial muscle cell, short arrow: submerged muscle cell). Actin filaments accumulate in several cells before mCherry becomes visible (**K,** arrows). **L-N** Columnar retractor muscle cells expressing mCherry. **O-Q** Optical longitudinal sections of primary polyps (13 d). Maximum projection of 53 layers. Numerous actin filaments are enriched in the outgrowing tentacle bulbs (**Q,** arrow). Tentacle longitudinal muscles have lost their connection to the apex of the epithelium and become situated at the base of the ectoderm, increasing their length with tentacle elongation (**P,** arrow).

The muscle system of the sea anemone *N. vectensis* can be roughly divided into a column and a tentacle system (Figure 1). The body column contains three morphologically and functionally distinct muscle groups. The parietal and a retractor muscle are longitudinal muscles. They are orientated along the oral-aboral axis of the polyp body column and located in different regions of the mesentery. The columnar ring muscle cells span the whole body column (Figure 1). Similarly, in the tentacle, ring and longitudinal muscles are present (Figure 1). With the exception of the tentacle longitudinal muscle, all muscles are of endodermal origin. The following subchapter describes in detail the two muscle systems in adults (Figures 2, 3, 4 and 5), as well as the development of the longitudinal muscles of the column (Figures 6, 7 and 8) and tentacle muscles (Figure 9).

### The body column and its muscles

Cross-sections of adult polyps revealed the presence of eight uniformly constructed mesenteries, each of which extends from the body wall to the actinopharynx. The typical architecture of a fully differentiated mesentery below the pharynx is shown (Figure 2A, Figure 3A). A characteristic bilateral rosette-like folding of the mesoglea is present at the base of each mesentery. These foldings are aligned by the myonemes of the parietal muscle cells (Figure 2B, Figure 3B). These myonemes connect directly to the neighboring ring muscle at the base of the mesentery (Figure 2D, Figure 3B) of the body column at both sides of the mesentery. Towards the distal part of the mesentery, the parietal muscle is separated from the retractor muscle by the stalk. In the retractor muscle the extensive folding of the mesoglea is even more pronounced and branched than in the parietal muscle, but it is formed only on one side (Figure 2C, Figure 3C). The side of the retractor muscle within the mesentery is non-random and defines the directive axis. Between the mesenterial filament (also termed septal filament) at the tip and the retractor muscle, a gametogenic region containing either eggs or sperm – directly embedded within the mesoglea – is present in sexually mature animals.Both parietal and retractor muscle are longitudinal muscles, with the former showing a more complex branching (secondary branching) of the mesoglea (Figure 2C, Figure 3C, Figure 4A); this increases the surface and enables more muscle cells to attach. As a result the overall surface area of myonemes is generally larger in the retractor than in the parietal muscle. In the ring muscle of the body wall the myonemes are arranged perpendicular to the oral-aboral axis. Instead of being a contiguous ring of muscle cells, it consists of eight portions situated between the mesenteries and merging directly into the parietal muscle to each side (Figure 2D, Figure 3B).

### Parietal and retractor muscles retain an epithelial organization

In cross-sections of the adult polyp, the cells of the retractor muscle are strongly entangled, making it difficult to follow each cell border from the base to the apex. At the proximal (towards the parietal muscle) and the distal border (towards the mesenterial filament) of the retractor muscle, the myonemes are closer to the apex of the epithelium. Here, a clear continuity can be observed, and the apical part of the cell contributes in building up the epithelium. Via thin cytoplasmic bridges, the apical part is connected to the robust basal, myofilament-containing part, which lies adjacent to the mesoglea (Figure 4E, F). The myonemes are large and well pronounced in the central region of the retractor muscle sheet (Figure 4G). Although not visible in single sections, these myonemes probably also still exhibit a connection to the epithelium. This interpretation is based on the fact that the space between the folded sheet of myonemes is filled with numerous cytoplasmic bridges, all of which originate from single myonemes running towards the apex of the epithelium. The myonemes are connected to each other by spot-like adherens junctions (Figure 4G) and are anchored to the mesoglea by focal adhesion sites (Figure 4E inlet).Likewise, the parietal muscle consists of a serial arrangement of myonemes (Figure 4B). The folding of the muscle sheets is less complex in this region, making it easier to follow individual cells from the base to the apex on single sections. Similar to the retractor muscle, the apical part of the cell in the parietal muscle is connected to neighboring cells to form an epithelium; the myonemes are connected to the apical part by cytoplasmic bridges (Figure 4D). A prominent neural plexus is situated on both sides of the parietal muscle; neurites run mainly along the oral-aboral axis (Figure 4C). The myonemes are again anchored by adherens junctions to neighboring cells and by focal adhesion sites to the mesoglea (Figure 4C, inlet 1).Ring muscle cells are oriented perpendicular to the longitudinal muscles. They span the body wall gastrodermis (adult endoderm) between the parietal muscle portions of all eight mesenteries and show a clear integration into the epithelium, having their myofilaments located at their bases (Figure 4H). In brief, all three muscle types of the body column show an epithelial organisation, but the retractor and parietal muscles are highly modified.

### The tentacle muscle system consists of basiepithelial myocytes and epitheliomuscular cells

Tentacles are basically tube-like expansions of the body column at the oral pole with continuation of the gastric cavity (Figure 1). Therefore they are structured in the same way as the double-layered body column, lacking any endodermal invaginations such as mesenteries in the body column (Figure 5A). Most of the cell types present in the tentacles are situated in the epidermis (adult ectoderm), making it most important for the functions of the tentacle. At least two different types of gland cells are present, one with electron-dense and one with electron-light vesicles. One of which has recently been shown to secrete a toxin [12]. Since the main function of the tentacle is to capture prey, it is unsurprising that it is very rich in spirocysts. The longitudinal muscle consists of a discontinuous sheet of myonemes located at the base of the epidermis, forming a ring around the mesoglea. A prominent neural plexus lies on top of that muscle layer; it also shows a ring-like structure (Figure 5B). To clarify whether the basally located myonemes exhibit a connection to the apex of the epithelium, as is the case in the columnar muscles, we produced serial longitudinal- and cross-sections of tentacles. We found that the nuclei of the muscle cells are located near the myofilaments and that no cytoplasmic bridges between the basal myonemes and the apical part of the epithelium are formed. The elongated muscle cells are arranged side by side, connected laterally by spot-like adherens junctions (Figure 5C + D). Through the discontinuous sheet of muscle cells, overlying epithelial cells send their processes and attach to the mesoglea (Figure 5C + F).The gastrodermis of the tentacle is mainly composed of circular muscle cells. They resemble the circular muscles of the body column in that their myofilaments are located basally and in that the connection between the apex and the base of their cells is not drawn into long and thin cytoplasmic bridges. Due to the low myofilament density, the tentacle ring muscle is comparatively weakly developed (Figure 5E).

### Development of columnar longitudinal muscles

To investigate the development of columnar longitudinal muscles, subpharyngeal cross-sections of different stages ranging from mid-planula to juvenile polyps were produced. Columnar longitudinal muscles are located in the mesentery, which corresponds to a folding of the endodermal epithelium. The first pair of mesenteries becomes visible on a macroscopic level at the primary polyp stage. They are called primary mesenteries and can be easily distinguished from the remaining three pairs of secondary mesenteries because of their early-formed mesenterial filaments at the distal tip. By comparison the secondary mesenteries form their mesenterial filaments only later during polyp growth. However, when reaching adulthood, all eight mesenteries have developed to a similar degree and they cannot be distinguished anymore on a macroscopic level.Needless to say, a mesenterial anlage is required before muscle cells can develop. A first morphological indication of muscle differentiation was observed in the primary mesenteries of the mid-planula stage (Figure 6A-C). The base of the mesentery (future parietal muscle site) lacks any clear signs of muscle cells (Figure 6B). At the same time, a few cells containing a small number of myofilaments were already visible along the mesentery, lying adjacently to the mesoglea (Figure 6C, inlet). Secondary mesenteries at this stage have not developed any morphologically visible muscular structures yet (data not shown). In late planula stages (Figure 6D-I) the future muscle regions become apparent. The retractor muscle cells of the primary mesenteries have formed additional myofilaments and are positioned side by side (Figure 6E). They emerge slightly distal of the mesentery base, already formed exclusively on one side of the mesentery. At this stage, no clear myofilament formation is visible at the base of the mesentery (Figure 6F). In secondary mesenteries, both muscle groups – parietal and retractor muscles – are identifiable. Unlike the situation in primary mesenteries, here the retractor muscle cells form at the tip of the mesentery, without any bias to one side yet (Figure 6G-I). Generally, muscle cells do not differentiate synchronously in all eight mesenteries. This process is usually quite variable, and neither primary, nor secondary mesenteries are distinctly ahead in the differentiation of muscle cells.In early primary polyps the morphology has changed noticeably. The body wall endoderm has become thinner and the secondary mesenteries are easier visible within the endoderm. The primary mesenteries no longer lie opposite each other and become clearly shifted to one side (Figure 7A). In the retractor- and parietal muscle region of primary and secondary mesenteries, the number of cells with myofilaments increases. At this stage the formation of a neural plexus next to the parietal muscle begins (Figure 7B). In contrast to the parietal muscle, retractor muscle cells of the primary mesenteries already start to constrict the myoneme from the rest of the cell, and cytoplasmic bridges become visible (Figure 7C). In secondary mesenteries (Figure 7D-F), myonemes of the retractor muscle are still located at the tip, but they are already more pronounced on one side of the mesentery (Figure 7F).In the juvenile polyp (Figure 7G-I) the relative position of the primary to the secondary mesenteries becomes clear (although already set in the early planula). The primary mesentery at this stage is composed of a distal mesenterial filament and a proximally located bulged tissue (extending from the stalk to the mesenterial filament) harbouring the retractor muscle at its base. In one of the six secondary mesenteries the mesenterial filament has started to form at the tip; in the remaining ones the retractor muscle is still the most distal structure (Figure 7G). In fully differentiated polyps the secondary mesenteries will have grown to the same size as the primary ones; then, the origin of every single mesentery can only be reconstructed by determining the position of the retractor muscles. The morphology of the parietal muscle resembles that of early stages with the exception that more cells are involved in building up the muscle sheet and that the myonemes become increasingly constricted from the apical epithelial part. The basal part maintains a connection to the apical one and neighboring muscle cells are anchored to each other by adherens junctions at their basal sides. In comparison to the situation in adults (Figure 2B, Figure 3B) the muscle sheets have not folded yet (Figure 7H). The myonemes of the retractor muscle are more densely packed with myofilaments than in the parietal muscle and the cytoplasmic bridge between the base and apex of the cell is already constricted. Like in the parietal muscle, the myonemes are connected to each other by adherens junctions. The muscle sheet is no longer arranged in a straight way, but has started to produce the first folding (Figure 7I). This process will proceed during the maturation of the juvenile, ultimately yielding the typically multiple-folded, one-sided muscle “flag”.The orientation of the retractor muscles leads to an internal bilateral symmetry and is consistent within different individuals. Primary mesenteries always have a predetermined place in this system. This makes it possible to predict the site of retractor muscle formation as soon as the mesenteries have set and before there are any morphological signs of muscle cell differentiation (Figure 7J).Staining with phalloidin additionally demonstrated the accumulation of F-actin in presumptive muscle cells during the development from the planula to the primary polyp. Endodermally located F-actin is already present in the early planula but does not yet show a clear concentration and uniform orientation (Figure 8A, D, G). In the late planula those actin threads become increasingly concentrated and exhibit a clear orientation along the oral-aboral axis. In the future tentacle bulb region, single cells have accumulated F-actin in their basal part (Figure 8B, E, H). In the early primary polyp an increasing number of future tentacle cells show concentrated F-actin; the columnar muscles already are differentiated into a parietal- and a retractor muscle region (Figure 8C, F, I).

### Development of tentacle longitudinal muscles

As mentioned above, fully differentiated longitudinal muscle cells of the tentacle are situated at the base of the epidermis and show no connection to the apex of the epithelium. In order to determine whether they emerge from originally epithelial cells, we used a transgenic line (MHC::mCherry) that expresses a fluorescent reporter gene (mCherry) under the control of a muscle-specific promotor (striated-*myhc*, formerly *myhc1*) specifically in retractor and tentacle muscles [13]. Expression of mCherry was first detected in 4 day mid-planula stages (Figure 9A-G). It starts with several single columnar cells at the oral pole close to the pharynx, spanning the whole thickness of the ectodermal epithelium. Small accumulations of actin filaments are already co-localized with the expression of mCherry in those cells (Figure 9B-D). At this stage most tentacle muscle cells have already abandoned their apical cell contacts and have sunken down to a basiepithelial position. At this stage of cellular differentiation their concentrated F-actin has increased (Figure 9E-G). Although most of the mCherry positive cells are already situated at the base, a few epithelial muscle cells may be present in early primary polyps. Many cells already formed spindle shaped, apical-basal orientated actin-filament accumulations at their bases before mCherry can be detected (Figure 9I-K). The expression of mCherry in the retractor muscle starts in the late planula (data not shown) and becomes more apparent in early primary polyps. In the latter, the nuclei and remaining cell bodies are visibly located on top of the actin-enriched contractile elements (Figure 9L-N). In older primary polyps (13d) the tentacle bulbs have started to grow out and thin actin-filaments have become aligned along the longitudinal axis of the tentacle (Figure 9O-Q). In comparison to earlier stages (7d) the orientation of actin-filaments has shifted by 90° from a basal-apical to a lateral character (Figure 9Q). Correspondingly, the muscle cells become spindle-shaped and oriented along the longitudinal axis of the tentacle, having lost their connection to the apical surface of the epithelium (Figure 9P).

## Discussion

**Figure 10:**
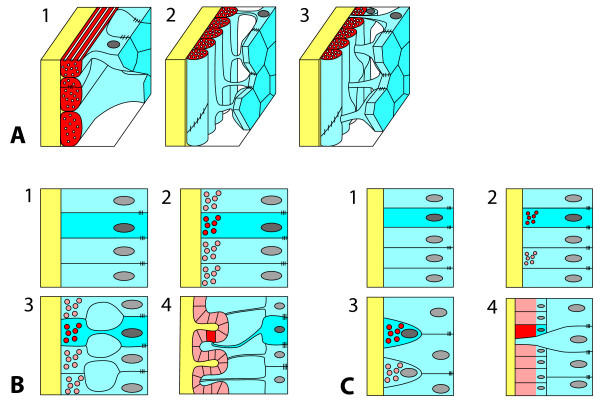
**Schematic representation of organization and development of muscle cells. A** Modes of muscle cell organization. The type 1 muscle cell corresponds to the classical epitheliomuscular cell. The cell is integrated into the epithelium by apical cell-cell junctions, connecting them to neighboring cells, while the basal part is elongated and houses myofilaments. Type 2 muscle cells are still epitheliomuscular; their connections between the apical and the basal part, however, are drawn out to form thin and elongated cytoplasmatic bridges. In type 3 muscle cells the apical cell junctions have been lost, resulting in a sinking to the base of the epithelium, with the nucleus situated near the myofilaments. **B + C** Development of type 2 & 3 muscle cells. In both types myofilaments start to become accumulated at the base of epithelial cells. **B** In type 2 muscle cells the apical cell-cell junctions remain and the connection between the apex and the base becomes constricted to form thin and elongated cytoplasmic bridges. The basally situated myonemes build up a continuous and folded muscle sheet. **C** Type 3 muscle cells lose their apical cell-cell junctions and sink down to the base of the epithelium, where they are arranged in a discontinuous muscle layer, enabling overlying epithelial cells to attach to the mesoglea. Color code: yellow: mesoglea, blue: epithelium, red: myofilaments, grey, nucleus.

In the present study we show that the muscular system of *N. vectensis* consists of two systems, both with a circular and at least one longitudinal portion. The muscle cells building up these two systems exhibit different levels of epithelial organization. Three main types can be distinguished: (1) The type 1 epitheliomuscular cell, in which the nucleus-containing part is connected to neighboring cells by apical cell-cell junctions, ensuring a complete integration into the epithelial cell complex. Their relatively weak myofilaments are housed in the elongated basal part, which lies adjacent to the extracellular matrix. This cell type is represented in the ring muscle cells of the column and tentacles (Figure 4H, Figure 5E, Figure 10A.1). This type is also found in Hydrozoa, which lack more elaborated muscle cell types. (2) The type 2 epitheliomuscular cell. It basically resembles type 1, with the difference that the apical-basal axis of the cell is extremely elongated, and that the apical and the basal part are connected only by an elongated and very slim cytoplasmic bridge. The longitudinal muscles of the column (parietal and retractor muscle) belong to this type (Figure 4D-F, Figure 10A.2). (3) The type 3 basiepithelial myocytes of the tentacle epidermis. In this type, the apical cell-cell junctions have been lost and the muscle cells are therefore positioned at the base of the epithelium. They constitute a spindle-shaped, elongated cell with a central thickening containing the nucleus. Like in other muscle types the myofilaments are still situated adjacent to the mesoglea (Figure 5C + D, Figure 10A.3).Type 2 and 3 represent specialized muscle cells and they underlie different courses of development. In both cases, myofilaments initially are formed at the bases of the cells. The type 2 muscle cell starts differentiating by constricting the basal from the apical part, maintaining a connection to neighboring cells by apical cell junctions. This process proceeds as the number of muscle cells increases, allowing the mesoglea and the basal part to undergo increasing folding of the continuous basal muscular sheet and the formation of elongated and slim cytoplasmic bridges, which connect the base to the apex (Figure 10B). Hence, the apical epithelial part does not have to follow the muscle folding or contraction. This arrangement allows maximal flexibility and mobility in the context of a one-cell layered epithelial organization. Type 3 muscle cells, in contrast, lose their apical cell junctions. This results in a localization at the base of the epithelium. Here, these cells form a discontinuous layer of myocytes (Figure 10C). Both types of muscle cells are anchored to the mesoglea basally. Type 1 and 2 muscle cells are classical epitheliomuscular cells. Type 3 muscle cells have lost their apical integration and therefore constitute basiepithelial myocytes.

### Biomechanical considerations

The three types of muscle cell formation presented here are interpreted to be a direct consequence of biomechanical constraints. Ring muscle cells are exclusively situated in the gastrodermis of either the column or tentacles. Beyond their function as contractile units they mainly serve to build up the epithelium. Ring muscle cells are the predominant cell type of that region. The epithelium is relatively flat and a long and thin cytoplasmic bridge is mechanically not necessary to maintain a connection between the base and the apex. In the longitudinal muscles of the column (parietal and retractor muscle) the situation is different. In both muscles the basally situated muscle sheet is pleated accordion-fashioned, enabling more myonemes to attach to the mesoglea; this increases the contractile strength per area [14]. As a result the surface area of the basal side of the epithelium considerably exceeds that of the apex. This requires modifying the interconnecting part if integration into the epithelium is to be maintained. This is exactly what we observed when examining the elongated and slim cytoplasmic bridges: they become progressively apparent when the muscle sheets start to fold.

Unlike in the column, longitudinal muscle cells of the tentacles are located in the epidermis. Tentacles serve to catch prey and are therefore rich in epidermal cell types such as gland cells and nematocytes, which facilitate this function. All cell types are more or less equally distributed in a ring-like manner around the mesoglea. This would leave less space for epitheliomuscular cells. For this reason we interpret the muscle cells to have lost their apical cell-cell junctions to acquire a basiepithelial position. As they have lost their apical cell junctions, the overlying cells must assume the role of building an epithelial barrier. In addition overlying cells require an anchoring to the mesoglea: this is now provided because the muscle layer is no longer continuous.

### Comparison of muscle cell types within Cnidaria

Different groups within the Cnidaria show a very high variability in terms of how and where contractile cells are formed. In hydropolyps the muscular system is exclusively composed of smooth epitheliomuscular cells. While longitudinally orientated myonemes are located in the epidermis, circularly arranged myonemes are located in the gastrodermis. Both epithelia mainly consist of epitheliomuscular cells [15]. In the hydromedusa of *Obelia* the ectodermal subumbrella is composed of epitheliomuscular cells with both radial helical myofibrils and submerged myocytes, which exhibit true striation [16]. In scyphopolyps the muscular system is exclusively formed by the ectoderm. Here the different muscle fields are physically continuous at the peristomial pits and are either epitheliomuscular (tentacle) or formed as myocytes (column) embedded within the mesoglea. Myofibrills are striated at least in some parts of the polyp before they merge into smooth fibers [17]. The muscle cells of Scyphomedusae are located in the ectodermal subumbrella and formed as circular striated and radial smooth epitheliomuscular cells [18,19]. Werner and colleagues have shown that the muscular system in cubopolyps mainly consists of ectodermal muscle cells [20]. They are either composed of myocytes, which are embedded into the mesoglea (*Carybdea*), or of myocytes and additional epitheliomuscular cells (*Tripedalia)*. With the exception of the tentacle tips, all muscle cells have smooth fibers. In Cubomedusae, muscles are subumbrellar (ectodermal) and epitheliomuscular with circular, striated fibers that turn radially in the frenulae of the velarium [21,3]. From anthozoan polyps, predominantly smooth and epitheliomuscular cells have been reported. Striated muscle fibers, however, have been found in a small subset of actiniarian anthozoans [22]. Hyman [23] and Muscatine & Lenhoff [14] report the existence of additional epidermal muscle cells that are independent from the supporting cells and have adopted a subepidermal position. The latter descriptions are not consistent with our findings because we demonstrate the myocyte-nature of longitudinal tentacle cells: they take on a basiepithelial position, connected on the basal side to the mesoglea. In fact they represent basiepithelial myocytes.

In summary, all groups within the Cnidaria have been described to possess ectodermally derived muscle cells, while endodermal ones are restricted to Anthozoa and hydropolyps. Hydropolyps seem to be the only group lacking striated muscle fibers. Note, however, that striated muscles have been documented only in single cases in Anthozoa, cubopolyps and scyphopolyps, and their absence is rather the rule. In general, the muscle fibers of most polypoid cnidarians are smooth, while medusae possess additional striated fibers [23]. Epitheliomuscular cells are present in all groups and myocytes at least in either the polyp or the medusa stage of every group (Table 1).

**Table 1:** Origin, striation and cellular organization of cnidarian muscles

	**Ectodermal**	**Endodermal**	**Striated**	**Smooth**	**Epitheliomuscular**	**Myocytes**
**Cubopolyp**	+	-	(+)	+	+	+
**Cubomedusa**	+	-	+	+	+	-
**Scyphopolyp**	+	-	(+)	+	+	+
**Scyphomedusa**	+	-	+	+	+	-
**Hydropolyp**	+	+	-	+	+	-
**Hydromedusa**	+	-	+	+	+	+
**Anthozoa**	+	+	(+)	+	+	+

“+”: trait present, “-“: trait absent, “(+)”: trait present, but only reported in single cases.

In general, cnidarian tissues show a low grade of division of labor [14]. It has been hypothesized that multifunctionality is a general feature of ancient cell types and that during the course of evolution these multiple functions were segregated among sister cell types. A case in point is epitheliomuscular cells [24]. Our observations support this hypothesis, as we were able to observe the process of de-epithelialization of initially epithelial muscle cells to a basiepithelial location in a single animal. In contrast, a comprehensive comparative analysis of the evolutionary origin of all known muscle proteins revealed that many components are either very ancient, pre-dating the origin of animals, or they evolved rather late in specific lineages [5]. No molecular synapomorphy has been found that would explain the emergence of striated muscles. This suggests that striated muscles have evolved independently in cnidarians, ctenophores and bilaterians, using a core set of ancient muscle proteins [5].

Cnidaria are still thought to exhibit the most primitive state of an interconnected muscular system [23]. The fact that both epitheliomuscular cells and myocytes are present in all cnidarian classes indicates that they must have already been present in the last common ancestor of Cnidaria and Bilateria. While in Cnidaria epitheliomuscular cells still play a major role in building up a contractile apparatus, this function is progressively adopted by mesodermal myocytes in Bilateria. Clearly, the evolution of mesoderm constitutes a crucial step in facilitating the emergence of complex muscular systems. Myocytes themselves, however, might have played a vital role in the early evolution of mesoderm. Nemertodermatid and acoel myocytes, like other mesodermal cells, apparently originate from the gastrodermis before they eventually emigrate into a subepithelial position [25]. A continuous sequence from epitheliomuscular cells to a subperitoneal musculature has been discovered in annelids and echinoderms, suggesting the epithelial organization of mesodermal muscle cells is an ancestral state [25-30].

In this context *N. vectensis* might represent an additional model for demonstrating the early stage of emigration of epitheliomuscular cells. *N. vectensis* myocytes have not been observed to entirely detach from the epithelium and to result in a mesenchymal, subepithelial position. Nonetheless, one can easily envisage such a process, which would ultimately lead to the situation in cubo- and scyphopolyps, where certain myocytes are completely embedded in the mesoglea [20,17].

## Competing interests

The authors declare that they have no competing interests.

## Authors’ contributions

SMJ carried out the experimental work, analyzed the data. MW contributed to the histological preparations, and critically evaluated the data and the ms. UT conceived of the study, analyzed the data and together with SMJ wrote the manuscript. All authors read and approved the final manuscript.

## References

[B1] Medina M Collins AG Silberman JD Sogin ML Evaluating hypotheses of basal animal phylogeny using complete sequences of large and small subunit rRNA Proc Natl Acad Sci U S A 2001 98 9707 9712 10.1073/pnas.171316998 11504944 PMC55517

[B2] Collins AG Phylogeny of Medusozoa and the evolution of cnidarian life cycles J Evol Biol 2002 15 418 432 10.1046/j.1420-9101.2002.00403.x

[B3] Gruner H Hartwich G Lehrbuch der Speziellen Zoologie. Band 1: Wirbellose Tiere. 2.Teil: Cnidaria, Ctenophora, Mesozoa, Plathelminthes, Nemertini, Entoprocta, Nemathelminthes, Priapulida 1984 Jena: Hrsg. von Hans-Eckhard Gruner. Bearb. von G. Hartwich. Gustav Fischer

[B4] Seipel K Schmid V Evolution of striated muscle: jellyfish and the origin of triploblasty Dev Biol 2005 282 14 26 10.1016/j.ydbio.2005.03.032 15936326

[B5] Steinmetz PRH Kraus JEM Larroux C Hammel JU Amon-Hassenzahl A Houliston E Woerheide G Nickel M Degnan BM Technau U Independent evolution of striated muscles in cnidarians and bilaterians Nature 2012 487 231 234 10.1038/nature11180 22763458 PMC3398149

[B6] Darling JA Reitzel AR Burton PM Mazza ME Ryan JF Sullivan JC Finnerty JR Rising starlet: the starlet sea anemone, *Nematostella vectensis* Bioessays 2005 27 211 221 10.1002/bies.20181 15666346

[B7] Genikhovich G Technau U The starlet sea anemone Nematostella vectensis: an anthozoan model organism for studies in comparative genomics and functional evolutionary developmental biology Cold Spring Harb Protoc 2009 doi:10.1101/pdb.emo129 10.1101/pdb.emo129 20147257

[B8] Technau U Steele RE Evolutionary crossroads in developmental biology: Cnidaria Development 2011 138 1447 1458 10.1242/dev.048959 21389047 PMC3062418

[B9] Frank P Bleakney JS Histology and sexual reproduction of the anemone *Nematostella vectensis* Stephenson 1935 J Nat Hist 1976 10 441 449 10.1080/00222937600770331

[B10] Fritzenwanker JH Technau U Induction of gametogenesis in the basal cnidarian *Nematostella vectensis* (Anthozoa) Dev Genes Evol 2002 212 99 103 10.1007/s00427-002-0214-7 11914942

[B11] Genikhovich G Technau U Induction of spawning in the starlet sea anemone *Nematostella vectensis*, in vitro fertilization of gametes, and dejellying of zygotes Cold Spring Harb Protoc 2009 doi:10.1101/pdb.prot5281 10.1101/pdb.prot5281 20147266

[B12] Moran Y Genikhovich G Gordon D Wienkoop S Zenkert C Ozbek S Technau U Gurevitz M Neurotoxin localization to ectodermal gland cells uncovers an alternative mechanism of venom delivery in sea anemones Proc Biol Sci 2012 279 1351 1358 10.1098/rspb.2011.1731 22048953 PMC3282367

[B13] Renfer E Amon-Hassenzahl A Steinmetz PRH Technau U A muscle-specific transgenic reporter line of the sea anemone, Nematostella vectensis Proc Natl Acad Sci U S A 2010 107 1 104 108 10.1073/pnas.0909148107 20018670 PMC2806778

[B14] Muscatine L Lenhoff HM Coelenterate biology. Reviews and new perspectives 1974 New York: Academic Press

[B15] Hess A Cohen AI Robson EA Observations on the Structure of Hydra as seen with the electron and light microscopes Q J Microsc Sci 1957 98 315 326

[B16] Chapman DM A new type of muscle cell from the Subumbrella of obelia J Mar Biol Ass 1968 48 667 688 10.1017/S0025315400019226

[B17] Chia F Amerongen HM Peteya DJ Ultrastructure of the neuromuscular system of the polyp of *Aurelia aurita* L., 1758 (Cnidaria, Scyphozoa) J Morphol 1984 180 69 79 10.1002/jmor.1051800108 30041506

[B18] Gladfelter WB Structure and function of the locomotory system of the Scyphomedusa *Cyanea capillata* Mar Biol 1972 14 150 160 10.1007/BF00373214

[B19] Anderson PAV Schwab WE The organization and structure of nerve and muscle in the jellyfish *Cyanea capillata* (Coelenterata; Scyphozoa) J Morphol 1981 170 383 399 10.1002/jmor.1051700309 30153719

[B20] Werner B Chapman DM Cutress CE Muscular and nervous systems of the cubopolyp (Cnidaria) Experientia 1976 32 1047 1049 10.1007/BF01933964

[B21] Satterlie RA Thomas KS Gray GC Muscle organization of the cubozoan jellyfish *Tripedalia cystophora* Conant 1897 Biol Bull 2005 209 154 163 10.2307/3593133 16260775

[B22] Amerongen HM Peteya DJ Ultrastructural study of two kinds of muscle in sea anemones: the existence of fast and slow muscles J Morphol 1980 166 145 154 10.1002/jmor.1051660203 30189712

[B23] Hyman L The invertebrates: Protozoa through Ctenophora 1940 New York: McGraw-Hill

[B24] Arendt D The evolution of cell types in animals: emerging principles from molecular studies Nat Rev Genet 2008 9 868 882 10.1038/nrg2416 18927580

[B25] Rieger RM Ladurner P The significance of muscle cells for the origin of mesoderm in bilateria Integr Comp Biol 2003 43 47 54 10.1093/icb/43.1.47 21680408

[B26] Rieger RM Über den Ursprung der Bilateria: Die Bedeutung der Ultrastrukturforschung für ein neues Verstehen der Metazoenevolution Verh Dtsch Zool Ges 1986 79 31 50

[B27] Rieger RM Lombardi J Ultrastructure of coelomic lining in echinoderm podia: significance for concepts in the evolution of muscle and peritoneal cells Zoomorphology 1987 107 191 208 10.1007/BF00312261

[B28] Westheide W Hermans CO The Ultrastructure of Polychaeta 1988 New York: Fischer

[B29] Stauber M The lantern of Aristotle: organization of its coelom and origin of its muscles (Echinodermata, Echinoida) Zoomorphology 1993 113 137 151 10.1007/BF00403091

[B30] Bartolomaeus T On the ultrastructure of the coelomic lining in the Annelida, Sipunculida and Echiura Microfauna Marina 1994 9 171 220

